# Supplementary material for: Flocking in complex environments—Attention trade-offs in collective information processing
Source: PLoS Comput Biol. 2020 Apr 6;16(4):e1007697. doi: 10.1371/journal.pcbi.1007697 (PMC7173936; doi:10.1371/journal.pcbi.1007697)
Supplement: S1 Text — Sections: I. Dynamical, directed interaction networks. II. Coordination-responsiveness trade-off as an emergent collective effect. III. Quantifying the coordination-responsiveness trade-off—global fitness function. IV. Modified model with higher priority of DS avoidance. V. Emergence of global order in the absence of informed individuals. VI. Discussion of idealizing model assumptions in relation to visual interactions. VII. Numerical implementation and experiments. (PDF) [file pcbi.1007697.s001.pdf]

SI Appendix
Supplementary Information
"Flocking in complex environments – Attention trade-offs in collective information processing"

Parisa Rahmani,[1,2] Fernando Peruani,[3] and Pawel Romanczuk[2,4,*]

[1]*Department of Physics, Institute for Advanced Studies in Basic Sciences (IASBS), Zanjan 45137-66731, Iran*
[2]*Institute for Theoretical Biology, Department of Biology, Humboldt Universität zu Berlin, Germany*
[3]*Université Côte d'Azur, Laboratoire J.A. Dieudonné, UMR 7351 CNRS, Parc Valrose, F-06108 Nice Cedex 02, France*
[4]*Bernstein Center for Computational Neuroscience, Berlin, Germany*

(Dated: April 10, 2020)

I. DYNAMICAL, DIRECTED INTERACTION NETWORKS

Network connectivity

The interaction network is directed and time dependent. For low attention capacity k, at any given time, it consists of a large number of small clusters (connected components), without links between them. Here, we are using the weak definition of connectedness for directed networks: A component of a network is connected if there exists at least one directed link between the corresponding agents. Throughout time these connected components are permanently reshuffled through continuous fission-fusion dynamics. With increasing k (or decreasing DS density) we observe a decrease of the number of connected components (Fig. S2a). Thus the total system consists of fewer but larger clusters. Eventually, for sufficiently high k, we have a finite probability over time to observe a fully connected network with a single connected component (Fig. S2b). The critical k at which fully connected networks can be observed increases with the density of DSs.

The disruption of local interaction networks, and therefore the fission-fusion dynamics, is strongly driven by response of individual agents to the distracting environmental cues. Here, we should emphasize that the interaction neighborhood of individual agents may be subject to significant fluctuations even in the absence of disrupting environmental cues. Due to stochastic motion, individuals can in principle switch their attention from one neighbor to another one, even if those neighbors are separated by a large spatial distance. However, this is not an exclusive feature of the kNN model. The same behavior can be also observed in metric models with a fixed interaction radius, where an agent may lose neighbors by moving away and instead gain new neighbors on the other side of its interaction zone. In metric models the maximal separation of such neighbor swaps is set by the diameter of the interaction zone. In topological models, this separation is in principle unbounded. But in heterogeneous environments with high density of DSs, we observe formation of dense agent clusters (see Supp. Videos). Hence, the maximal separation in attention swaps is effectively limited by the typical self-organized cluster size.

We measured the average life time of individual network edges as a function of DS density ρ_{DS} and attention limit k. With increasing DS density (or decreasing k) we observe also a decrease in the average life time of an edge between two interacting agents (Fig. S2c). Thus, overall, with increasing DS density or decreasing k the (local) interaction networks become more dynamic.

Stationary distribution of in and out-degrees

From our numerical simulations, we can extract the stationary probability distribution $f(D_{out}, D_{in})$ for an agent having a particular combination of out-degree D_{out} and in-degree D_{in}.

In homogeneous environments, $\rho_{DS} \to 0$, the out-degree of each agent is directly set by the attention capacity k. Thus, the distribution of individual in and out-degrees $f(D_{\rm out}, D_{\rm in})$ is sharply peaked at $D_{\rm out} = k$. The in-degree is not fixed: Whereas a focal individual i pays attention only to k nearest neighbors, the number of other agents paying attention to it may be lower or higher. Therefore, we observe a spreading out of $f(D_{\rm out}, D_{\rm in})$ along the in-degree axis.

* pawel.romanczuk@hu-berlin.de

This pattern holds also for finite number of DSs, however, now in addition, there is also a finite probability that an agent interacting with a DS ignores its neighbors (see Fig. 2c,d). In this case, its out-degree with respect to social interactions is zero: $D_{\mathrm{out}} = 0$, while the in-degree can assume many possible values depending on how many other agents pay attention to it at a given time. In general, for a finite DS density, the combined distribution $f(D_{\mathrm{out}}, D_{\mathrm{in}})$ shows a bimodal distribution with two maxima at $D_{\mathrm{out}} = 0$ and $D_{\mathrm{out}} = k$.

For low k, the fraction of agents responding to DSs remains low even at very high ρ_{DS}. The average out-degree as well as the average in-degree increases as expected with increasing k. However, the expected in-degree of direct responders with $D_{out} = 0$ is always lower then the expected in-degree of other agents with $D_{out} > 0$. As a results, for low k ($k < 3$), DS responders become often completely isolated from other agents by having $D_{in} = D_{out} = 0$.

II. COORDINATION-RESPONSIVENESS TRADE-OFF AS AN EMERGENT COLLECTIVE EFFECT

In order to show that the observed effects are genuinely collective and can not be traced back only to the behavior of informed agents we compare the results of interacting agents with a non-interacting system, in which the strength of social interactions is set to zero ($\gamma_s = 0$). Like in the model discussed in the main text, the attention slots of agents are restricted to k; agents only pay attention to DSs which are among their kNO and within a distance smaller than the radius of repulsion, but do not react to other agents which they perceive. Fig. S3a clearly shows that in the absence of interaction for all values of k the accuracy of the flock is low. However, if we introduce interaction to the system, the collective accuracy increases significantly for all k. For low k values we always observe the largest increase, whereas at high k values and in particular high DS density we observe the smallest increase.

If we consider the accuracy of informed and uninformed individuals separately, we observe a qualitatively different behavior: On the one hand, uninformed individuals move randomly in the absence of interactions (vanishing accuracy), while with finite social interactions they exhibit an accuracy of $C \approx 0.8$ at $k = 2$, which then decreases continuously with increasing k (Fig. S3b). As the ratio of informed individuals is $R_{inf} = 0.1$, the accuracy of only uninformed agents exhibits only a small difference to the total accuracy (Fig. S3a). On the other hand, in the absence of social interactions, informed individuals have high accuracy at low k, which exhibits initially a moderate decrease with increasing k, and then asymptotes to a constant, DS density-dependent, value. Therefore the behavior of only informed agents is qualitatively different from the behavior of the total system (Fig. S3c). We note the overall increase in the accuracy of informed individuals in the social model in comparison to the non-social model, which is due to social interactions with uninformed individuals. Here, uninformed individuals may be viewed as an information "reservoir": Informed individuals bias the motion of uninformed individuals through social interactions. If single informed individuals get distracted by environmental cues, the social interactions with their neighbors who keep the memory of the consensus direction allows a quicker alignment with the preferred direction of motion.

III. QUANTIFYING THE COORDINATION-RESPONSIVENESS TRADE-OFF – GLOBAL FITNESS FUNCTION

We can quantify the emergent trade-off between coordination and responsiveness by introducing the following global fitness function depending on the collective accuracy C and DS avoidance A:

$$\tilde{F}(C, A, k) = b_c C + b_a (A - 1) \tag{1}$$

with b_c being the benefits per unit of directional consensus, and b_a the benefits per unit of DS avoidance. Without loss of generality, the equation can be rescaled by b_c to

$$F(C, A) = C + \beta(A - 1), \tag{2}$$

where $\beta = b_a/b_c$ represents the relative benefits of DS avoidance versus coordination. Please note that F is not fitness in a strict evolutionary sense (selection at the level of individuals), as it is a function of collective variables A and C. Here, "fitness" refers rather to a collective utility function, where (local) maxima correspond to (local) optimal collective strategies for homogeneous collectives. For a given set of parameters, A and C are calculated in a stationary regime of a stochastic system. Thus both variables reflect the average ability of individuals to avoid DSs and to coordinate with their neighbors in homogeneous groups.

For $\beta = 1$ both behaviors yield equal benefits, whereas for $\beta > 1$ ($\beta < 1$) DS avoidance is more (less) beneficial than coordination. For a wide range of DS densities and angular noises, we observe two distinct maxima of F in the β, k-plane. For $\beta \ll 1$ coordination is much more beneficial than DS avoidance and the maximum of F is located at small k where coordination is highest. With increasing β this maximum decreases and we eventually see a rise

of a second maximum at large k, leading to two local maxima at intermediate β. Eventually, for large β, where DS avoidance becomes far more important than coordination, we observe a single global maximum at large k.

IV. MODIFIED MODEL WITH HIGHER PRIORITY OF DS AVOIDANCE

In our model, we weight the attention to social cues and environmental cues in the same exact way: Agents may only respond to a cue if it is within the agent's k-nearest objects (kNO). Thus individuals can only interact with distraction sites if their distance rank is within their attention capacity. In other words, due to limited attention capacity an individual whose attention is saturated with social cues, may not detect a DS even if it is within the corresponding repulsion zone. It can be interpreted as the natural consequence of the attention of individuals being distracted by other (social) cues. However, if the DSs signal danger, it is natural to assume that individuals are more sensitive to environmental cues than to social cues, i.e. that individuals put a higher attention weight to distraction sites than to their neighbors.

In order to demonstrate that our general findings are present even if there is a higher effective attention weight assigned to the environmental cues, we devised a simple model extension where the attention to nearby objects is allocated differently putting a higher priority on DS detection. In the extended model, we introduce an additional parameter P_{direct}, which represents the probability that the agent will detect the DS once it is within the avoidance zone, independently of its social neighborhood, i.e. independently whether the nearby DS is within its kNO. For $P_{direct} = 0$ the extended model reduces to the original model assuming equal attention weights of social and environmental cues, as discussed in the main text. For $P_{direct} = 1$ the agents deterministically detect the DS once they are within the avoidance zone. Our qualitative result on the attention-responsiveness trade-off remains clearly valid as long as $P_{direct} < 1$ (see Fig. S6). Thus as long as there is some sensory "interference" between the response to environment and response to social cues, which is reasonable to assume for agents with limited sensory and cognitive capability using the same sensory system (e.g. vision) to interact with both, there will be a corresponding trade-off. This shows that the observed trade-off is indeed a fundamental emergent property, not dependent on our simplifying model assumption of equal attention weights to social and non-social cues.

.

V. EMERGENCE OF GLOBAL ORDER IN THE ABSENCE OF INFORMED INDIVIDUALS

In the absence of informed individuals $R_{inf} = 0$, there is no preferred direction of motion. Here, instead of collective accuracy, coordination can be quantified using average polarization of the flock

$$\tilde{C} = \frac{1}{N} \left\langle \sum_{i=1}^{N} \hat{u}_i \right\rangle , \tag{3}$$

which is equivalent to the ferromagnetic order parameter in physics. By replacing C by $\tilde{C}$, we observe the same fundamental coordination-responsiveness trade-off as discussed in the main text (Fig. S7).

VI. DISCUSSION OF IDEALIZING MODEL ASSUMPTIONS IN RELATION TO VISUAL INTERACTIONS

For computational efficiency we considered in our work kNN-interactions (here kNN refers to interaction with k-nearest neighbors). However, we also tested the Voronoi variant of the nearest-neighbor interaction (see SI Figure S8), which is closely related to the so-called balanced kNN-network discussed in [1].

In order to keep the model as simple as possible we refrained from considering finite size agents and distraction sites (DSs). This would require a whole new set of modeling decisions e.g. on the actual physical interaction of agents and finite size DSs in addition to repulsion zones around them (DSs as "obstacles" with excluded volume). Hence, we explicitly assume point-like agents and point-like environmental cues, which are surrounded by danger zones which themselves represent the areas where an agent responding to the cue attempts to move away from it as fast as possible. Line of sight obstructions are mathematically speaking not possible in this model. This idealizing assumption is reasonable in cases where the actual physical dimensions of the agents and environmental cues are small compared to the typical interaction ranges. Furthermore, we consider here, for simplicity, a two-dimensional system which in itself is an idealization. Real collective behavior in nature happens in three dimensions whether its aquatic systems (e.g. fish schooling) or terrestrial systems (e.g. ungulate herds). In 3D, even for finite size objects

complete blocking of the line of sight becomes much harder, especially if we consider not too dense environments, e.g. individuals can look above or through suspicious grass or smaller bush structures. Furthermore, for the kNN interaction network, the rank of nearest objects is intrinsically related to their distance, and in particular for high k we observe the formation of dense groups. Thus, the situation where one of neighboring agents is within the kNO set and at the same time "behind" a distraction site is first very rare, and second only short-lived due to the flocking dynamics. In our work, we also considered a Voronoi variant of our model, which corresponds to a spatially balanced kNN-model, where obstructions of the "line of sight" become even less likely. Here, the qualitative results remain absolutely unchanged (see SI Figure S8). Therefore, taking care of such rare line-of-sight obstructions does not change the main findings of our paper on the coordination-responsiveness trade-off.

We note that the Voronoi networks were shown also to provide a good approximation of visual networks in 2D which explicitly account for occlusions [2]. Calculation of visual networks as in [2] relies on ray-casting and is computationally prohibitively expensive, especially for long simulations of large flocking systems in complex environments, as considered here. Thus, in order to be able to investigate systematically the self-organized collective behavior in large systems for broad range of parameters, we constrained ourselves here to the kNN and kNN-Voronoi interaction networks.

VII. NUMERICAL IMPLEMENTATION AND EXPERIMENTS

The mathematical model was implemented in C/C++ with the k nearest object interaction based on the kd-tree structure from the CGAL library [3]. The kNN interaction implementation with periodic boundary conditions was combining the standard kd-tree based algorithm with generation of mirror images of the environment to account for the interactions on a torus.

The stochastic equations of motion were numerically integrated using a standard Euler-Maruyama scheme [4]. The numerical time step was set to $dt = 0.1$, whereby also smaller time steps were tested to show that the general results do not depend on the time step.

All the data were obtained by averaging over 20 realizations, each including 5×10^4 relaxation time steps and 10^5 stationary time steps.

[1] M. Camperi, A. Cavagna, I. Giardina, G. Parisi, and E. Silvestri. Spatially balanced topological interaction grants optimal cohesion in flocking models. *I*nterface Focus 2, 6, 2012.

[2] A. Strandburg-Peshkin, C.R. Twomey, N.W.F. Bode, A.B. Kao, Y. Katz, C.C. Ioannou, S.B. Rosenthal, C.J. Torney, H.S. Wu, S.A. Levin, I.D. Couzin. Visual sensory networks and effective information transfer in animal groups. *C*urrent Biology 23, 17, 2013.

[3] M. Bäsken. 2D Range and Neighbor Search. In CGAL User and Reference Manual. *C*GAL Editorial Board, 4.13 edition, 2018

[4] R. Mannella. Integration of stochastic differential equations on a computer. *I*nternational Journal of Modern Physics C 13(09) 1177-1194, 2002.

SUPPLEMENTARY VIDEOS

Each supplementary movie shows the spatial dynamics in the main panel (left), the corresponding accuracy C versus time (top right) and the corresponding DS avoidance A versus time (bottom right). The black line at $A = 1$ corresponds to the DS avoidance of non-interacting agents (see Materials and Methods). The first part shows the initial development of the system ($t = 0 - 200$), the second part shows the stationary state at large times ($t = 5200 - 5400$).

Supplementary Video 1: Collective behavior at high density of DSs for low attention capacity $k = 1$ characterized by high accuracy of collective migration.

Supplementary Video 2: Collective behavior at high DS densities for high attention capacity $k = 24$ characterized by efficient response to environmental cues.

Supplementary Video 3: Collective behavior in structured environment with a circular DS-free region for low attention capacity $k = 1$.

Supplementary Video 4: Collective behavior in structured environment with a circular DS-free region for high attention capacity $k = 24$.

SUPPLEMENTARY FIGURE 1

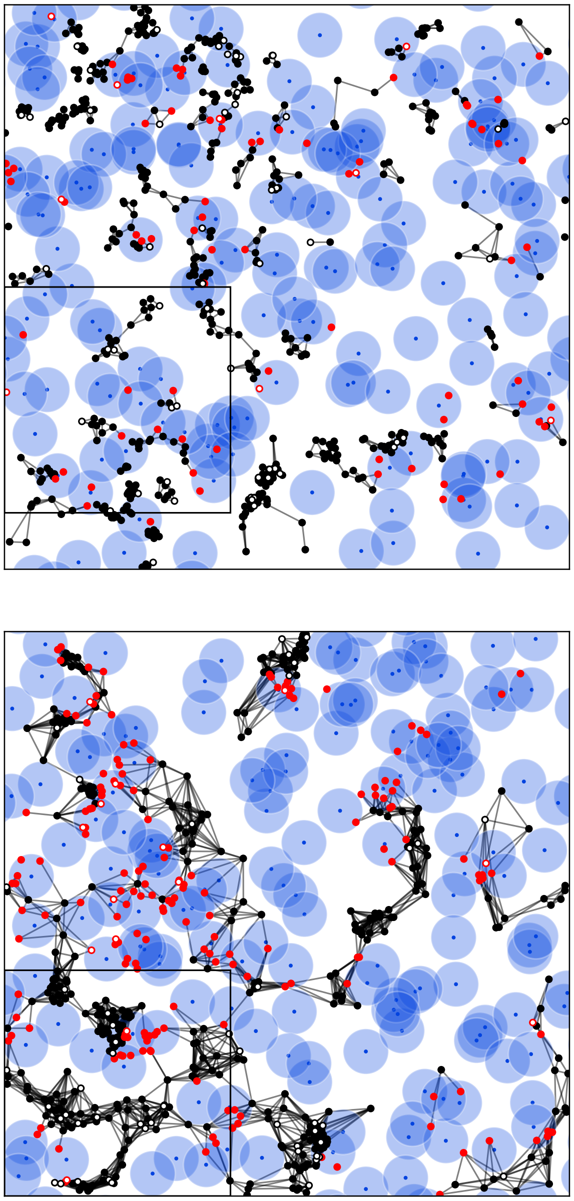

FIG. S1. Snapshots of the (undirected) social interaction network in random environments with $\rho_{DS} = 0.25$ for $k = 3$ upper panel, and $k = 12$ lower panel, at $R_{inf} = 0.1$. Black agents are socially interacting, and red agents react to DSs. Informed and uninformed individuals are represented by empty and filled circles, respectively. Light blue circles are repulsion zones of DSs specified with blue dots. For the sake of clarity, the links between agents interacting with their periodic neighbors are removed. The black squares depict the close-ups shown in panels a, b of Fig. 2 (main text). For low attention capacity ($k = 3$) the network is sparse, composed of many small connected components, whereas for large attention capacity ($k = 12$), the network is highly connected with less components.

SUPPLEMENTARY FIGURE 2

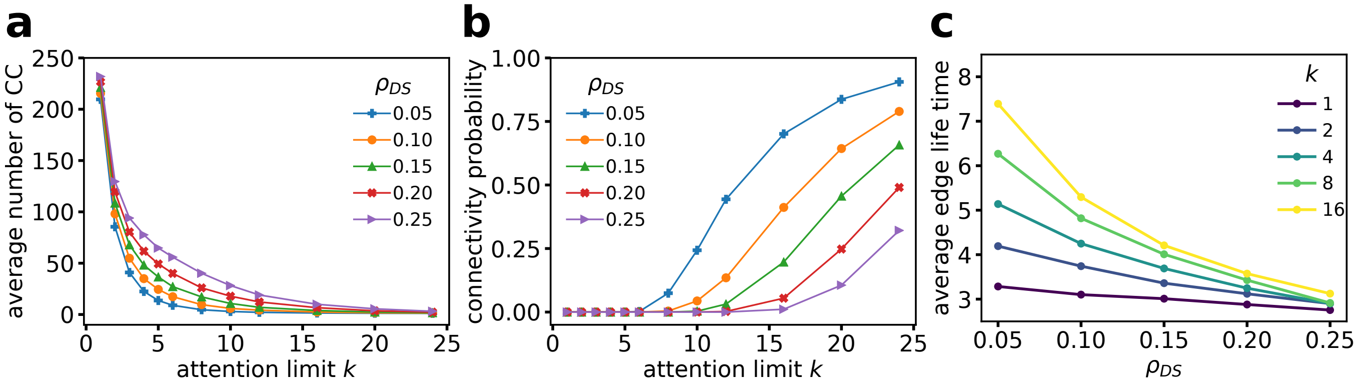

FIG. S2. Temporal interaction networks. **a**: Average number of connected components (CC) of the interaction network versus attention limit k. For all DS densities ρ_{DS}, we observe a fast decay of the number of connected components, which due to constant number of agents N is equivalent to the growth of the average connected component size, indicating a more tightly connected temporal network. **b**: The probability of observing one connected component during simulation. By increasing DS density, nonzero probability happens at larger k values. **c**: The average life time of an edge in the interaction network decreases with increasing density of DSs. However with increasing k for a fixed DS density, we observe longer life times due to increased connectivity in the interaction network.

SUPPLEMENTARY FIGURE 3

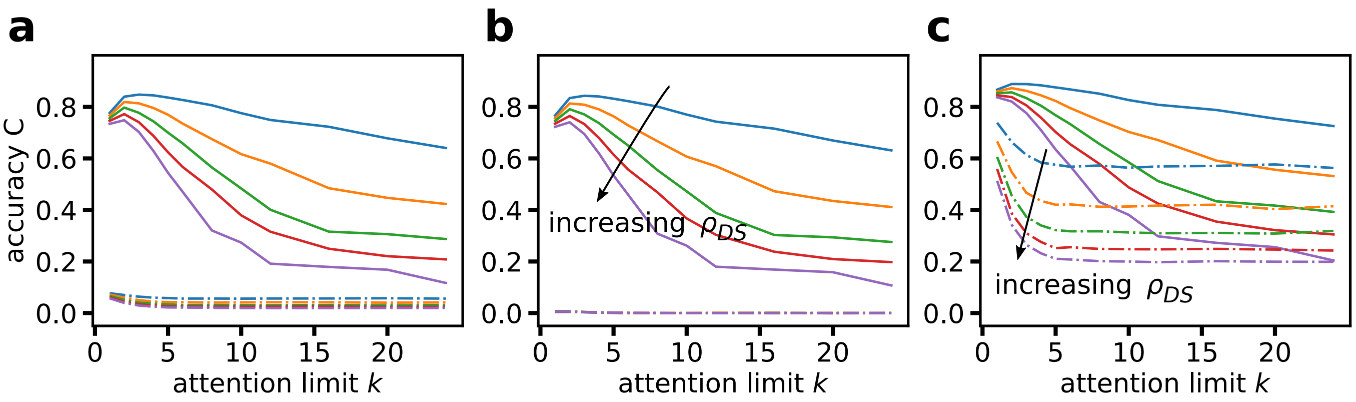

FIG. S3. Emergent collective behavior. Accuracy C vs attention limit k calculated for the whole system (**a**), uninformed individuals (**b**) and informed individuals (**c**). Solid lines are for the interacting system and dashed dotted lines are for the non-interacting system with $\gamma_s = 0$.

SUPPLEMENTARY FIGURE 4

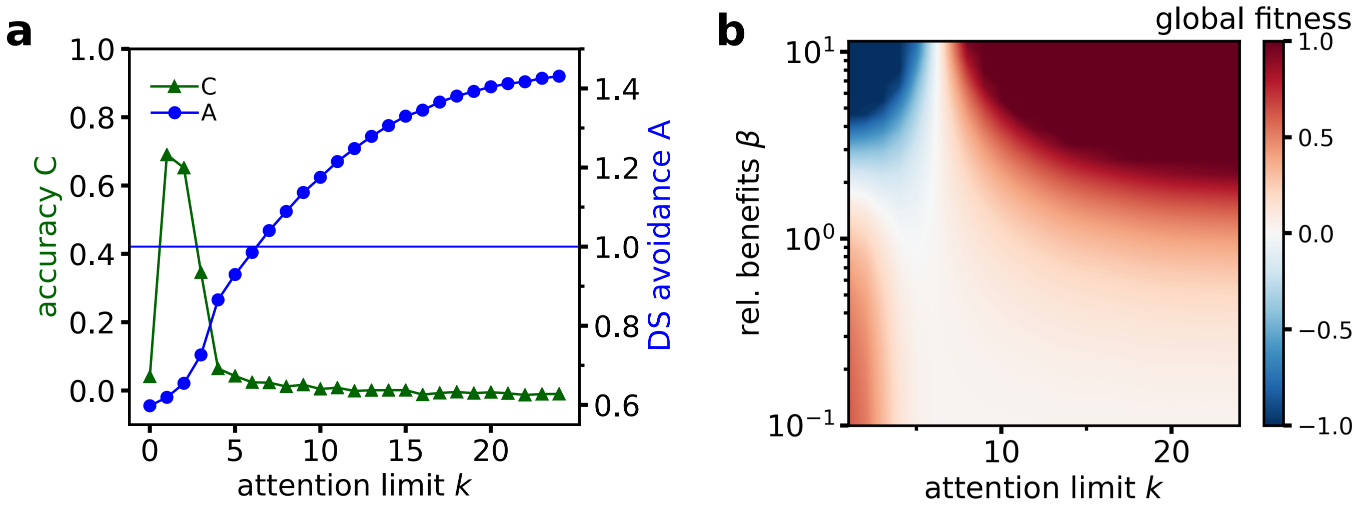

FIG. S4. Collective accuracy and DS avoidance in a structured environment with circular DS-free path. **a**: Accuracy C (triangles) and normalized DS avoidance A (circles) versus attention limit k. The horizontal line, $A = 1$, corresponds to DS avoidance of non-interacting agents. For socially interacting agents with low k values ($k = 1, 2$), we observe high accuracy C together with almost complete ignorance towards environmental cues. By increasing k, more agents start to sense the environment and react to DSs. At high k, the collective behavior is fully determined by the local environmental features: We observe collective rotation along the circular path and complete ignorance of the global migration direction accessible to informed individuals (see Supp. Movie S4). This trade-off is shown quantitatively by the global fitness function in panel **b** versus attention capacity k and relative DS avoidance benefit β. There are two maxima in global fitness, one for low k, $\beta \ll 1$, showing migration accuracy to be beneficial for the group, the other at high k, and $\beta > 1$, which indicates higher benefits associated with DS avoidance in comparison to collective accuracy.

SUPPLEMENTARY FIGURE 5

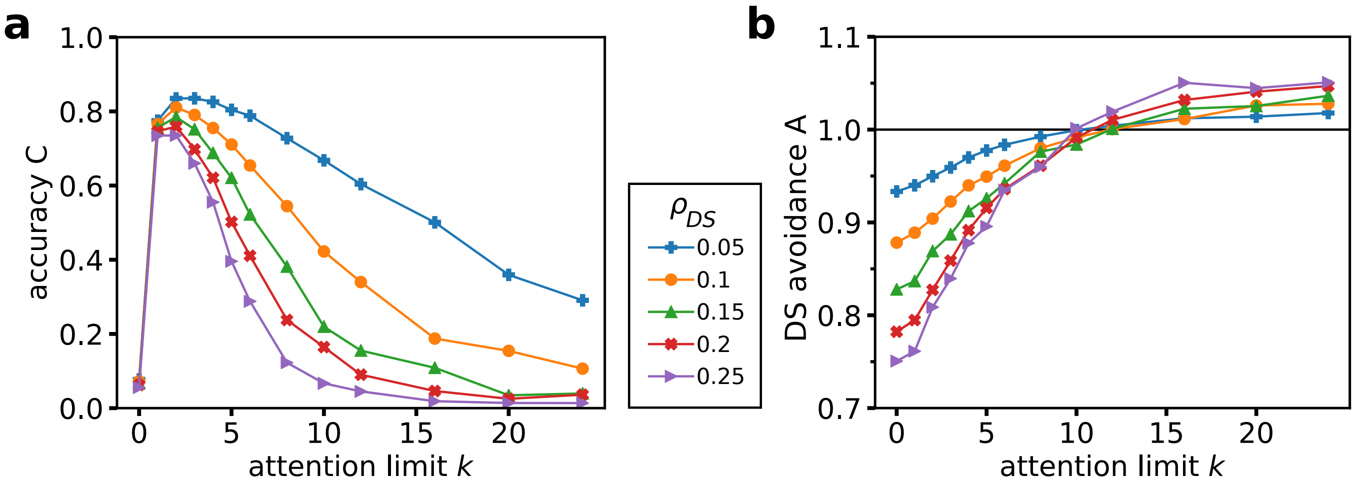

FIG. S5. Attention trade-off in a group of agents with active signalers. Each agent connected to another individual signalling direct interaction with a DS (direct responder), pays only attention to the signaller(s) and ignores other social cues. Accuracy C (**a**) and DS avoidance A (**b**) versus attention limit k for different DS densities at $R_{inf} = 0.1$.

SUPPLEMENTARY FIGURE 6

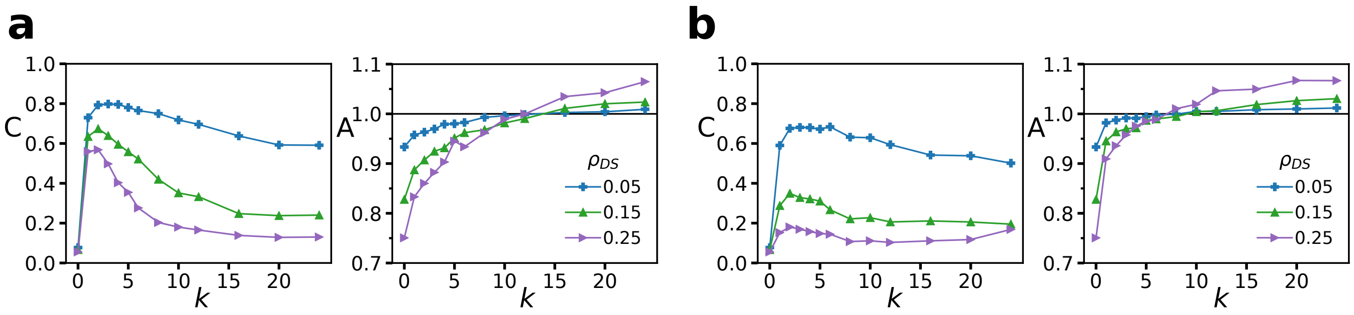

FIG. S6. Model extension with the priority of DS avoidance. Accuracy C and DS avoidance A vs attention limit k in a model where the agents first detect DSs with some probability and otherwise interact with their kNO. **a**: $P_{direct} = 0.2$, **b**: $P_{direct} = 0.5$.

SUPPLEMENTARY FIGURE 7

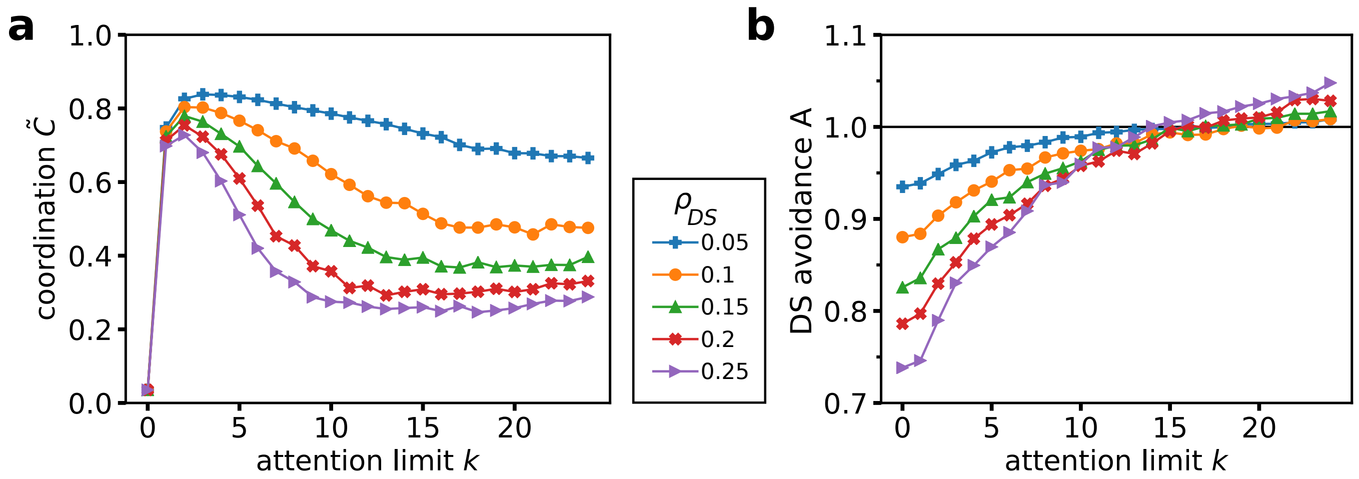

FIG. S7. Emergence of global order in the system with no informed individuals, $R_{inf} = 0$. **a**: Coordination $\tilde{C}$ (directional order) versus attention limit k for different DS densities. **b**: DS avoidance versus attention limit k. $A = 1$ corresponds to non-interacting agents. The qualitative behavior with a coordination-responsiveness trade-off is similar to the model with informed individuals, but here instead of a specific direction, the emergent consensus direction is random (spontaneous symmetry breaking).

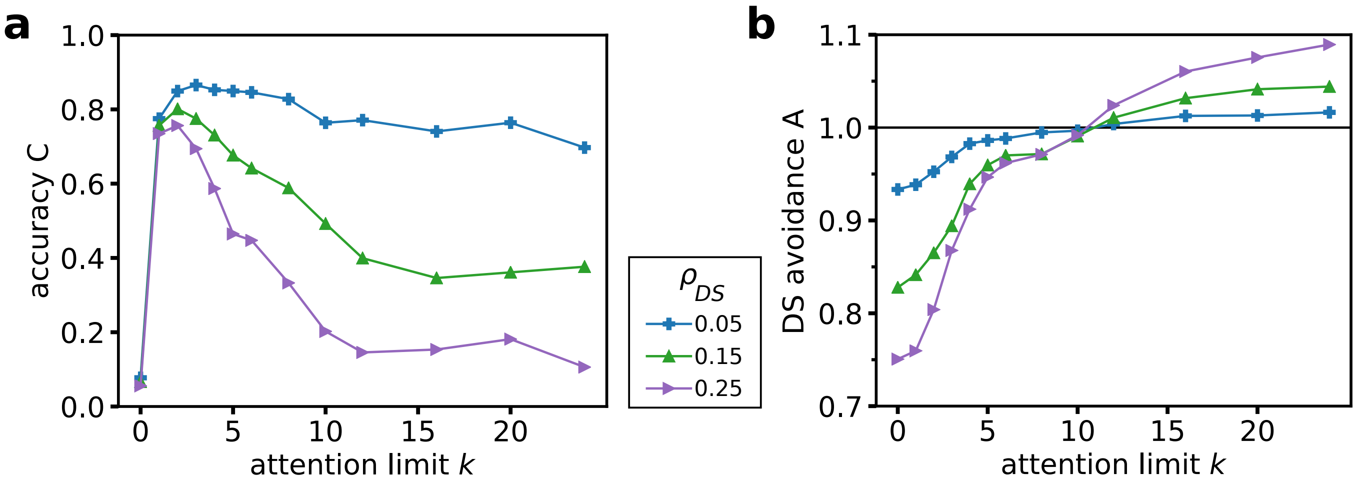

FIG. S8. Collective motion of agents with Voronoi-based kNN interaction network. Here, k nearest agents are selected from first shell of Voronoi neighbors. If the number of neighbors in first layer is smaller than k, then depending on k, the second Voronoi shell is considered. It is defined by the Voronoi neighborhood of the (direct) Voronoi neighbors of the focal agent. Accuracy C (**a**) and DS avoidance A (**b**) versus attention limit k at $R_{inf} = 0.1$.

SUPPLEMENTARY FIGURE 9

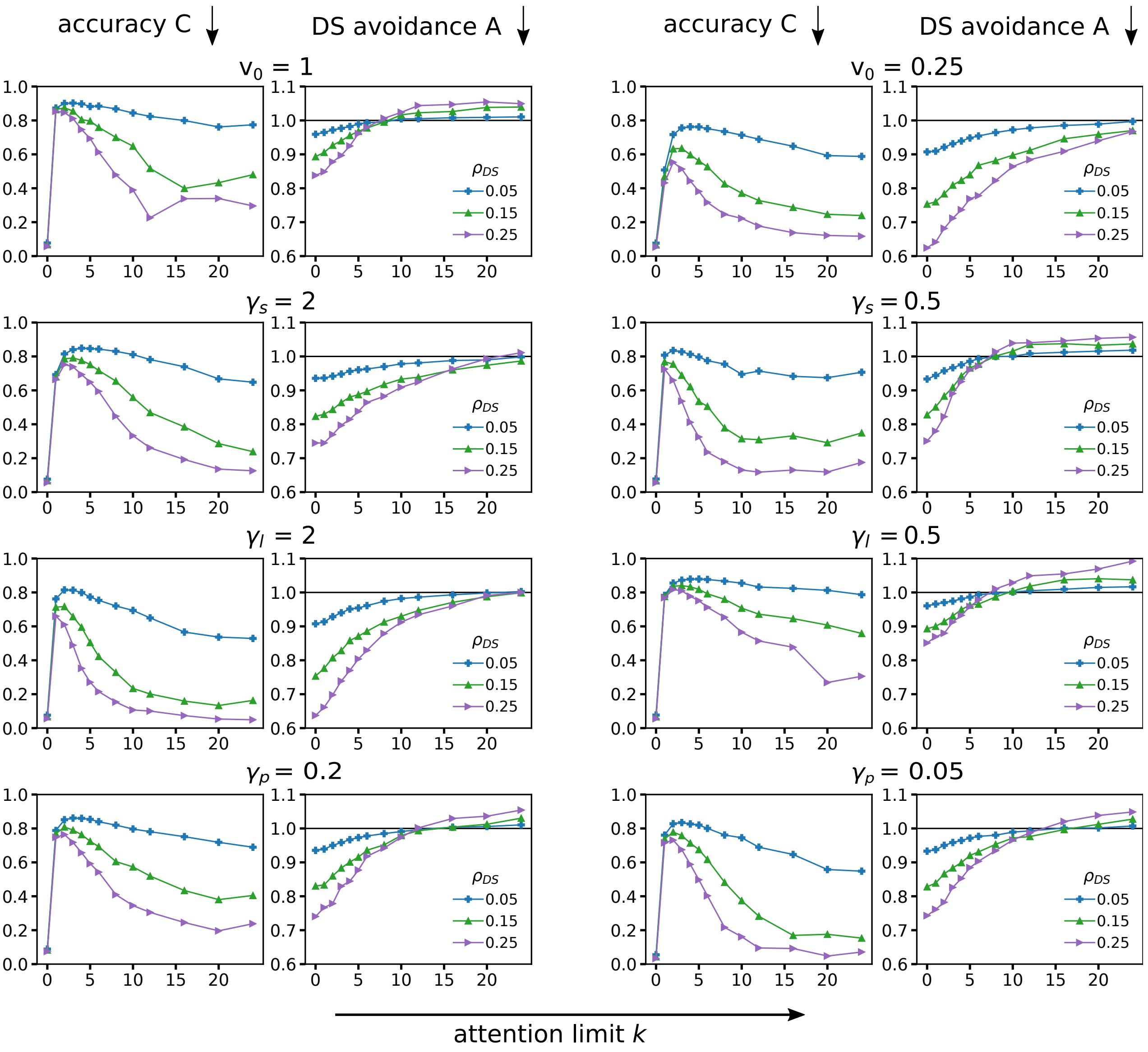

FIG. S9. Robustness of the general results for C and A vs k with respect to variation of model parameters. The panels show results for changing individual model parameters by a factor of 2 (left columns) and 0.5 (right columns). Different rows represent variation of different parameters, from top to bottom: v_0, γ_s, γ_l, γ_p. The non-varied parameters are always set to the default parameters: $v_0 = 0.5$, $\gamma_s = 1$, $\gamma_l = 1$, $\gamma_p = 0.1$.
