# Supplementary material for: Flocking in complex environments—Attention trade-offs in collective information processing
Source: PLoS Comput Biol. 2020 Apr 6;16(4):e1007697. doi: 10.1371/journal.pcbi.1007697 (PMC7173936; doi:10.1371/journal.pcbi.1007697)
Supplement: S1 Fig — Snapshots of the (undirected) social interaction network in random environments with ρDS = 0.25 for k = 3 upper panel, and k = 12 lower panel, at Rinf = 0.1. Black agents are socially interacting, and red agents react to DSs. Informed and uninformed individuals are represented by empty and filled circles, respectively. Light blue circles are repulsion zones of DSs specified with blue dots. For the sake of clarity, the links between agents interacting with their periodic neighbors are removed. The black squares depict the close-ups shown in panels a, b of Fig 2 (main text). For low attention capacity (k = 3) the network is sparse, composed of many small connected components, whereas for large attention capacity (k = 12), the network is highly connected with less components. (PDF) [file pcbi.1007697.s006.pdf]

SUPPLEMENTARY FIGURE 1

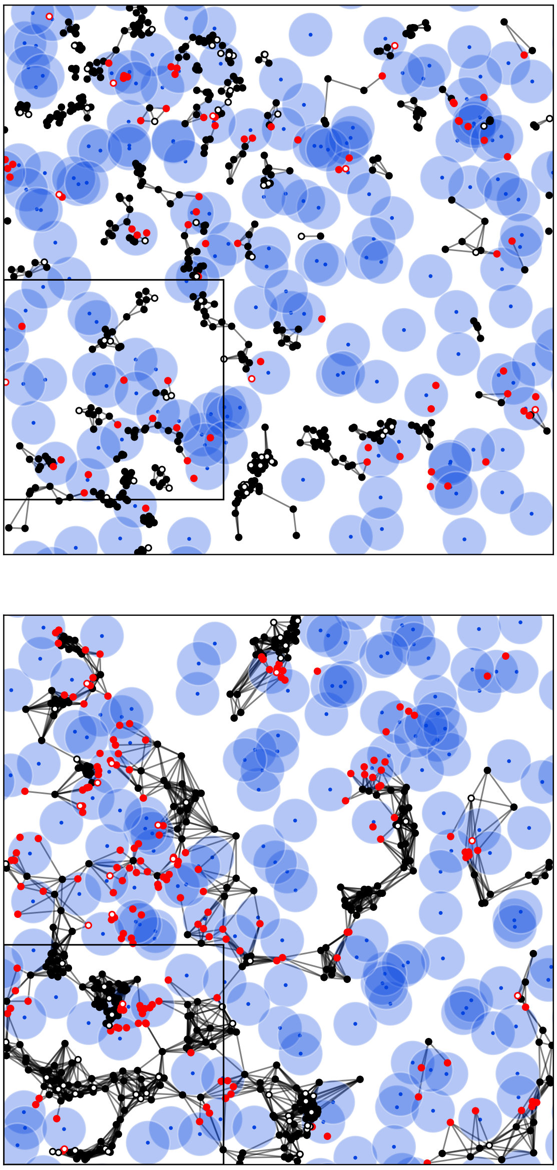

FIG. S1. Snapshots of the (undirected) social interaction network in random environments with $\rho_{DS} = 0.25$ for $k = 3$ upper panel, and $k = 12$ lower panel, at $R_{inf} = 0.1$. Black agents are socially interacting, and red agents react to DSs. Informed and uninformed individuals are represented by empty and filled circles, respectively. Light blue circles are repulsion zones of DSs specified with blue dots. For the sake of clarity, the links between agents interacting with their periodic neighbors are removed. The black squares depict the close-ups shown in panels a, b of Fig. 2 (main text). For low attention capacity ($k = 3$) the network is sparse, composed of many small connected components, whereas for large attention capacity ($k = 12$), the network is highly connected with less components.
