# Supplementary material for: Flocking in complex environments—Attention trade-offs in collective information processing
Source: PLoS Comput Biol. 2020 Apr 6;16(4):e1007697. doi: 10.1371/journal.pcbi.1007697 (PMC7173936; doi:10.1371/journal.pcbi.1007697)
Supplement: S2 Fig — a: Average number of connected components (CC) of the interaction network versus attention limit k. For all DS densities ρDS, we observe a fast decay of the number of connected components, which due to constant number of agents N is equivalent to the growth of the average connected component size, indicating a more tightly connected temporal network. b: The probability of observing one connected component during simulation. By increasing DS density, nonzero probability happens at larger k values. c: The average life time of an edge in the interaction network decreases with increasing density of DSs. However with increasing k for a fixed DS density, we observe longer life times due to increased connectivity in the interaction network. (PDF) [file pcbi.1007697.s007.pdf]

SUPPLEMENTARY FIGURE 2

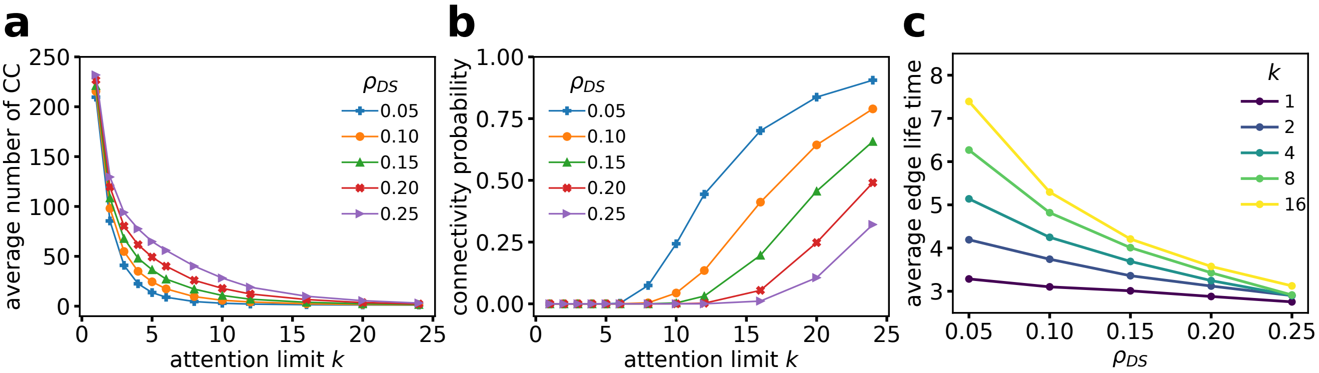

FIG. S2. Temporal interaction networks. **a**: Average number of connected components (CC) of the interaction network versus attention limit k. For all DS densities ρ_{DS}, we observe a fast decay of the number of connected components, which due to constant number of agents N is equivalent to the growth of the average connected component size, indicating a more tightly connected temporal network. **b**: The probability of observing one connected component during simulation. By increasing DS density, nonzero probability happens at larger k values. **c**: The average life time of an edge in the interaction network decreases with increasing density of DSs. However with increasing k for a fixed DS density, we observe longer life times due to increased connectivity in the interaction network.
