# Supplementary material for: Flocking in complex environments—Attention trade-offs in collective information processing
Source: PLoS Comput Biol. 2020 Apr 6;16(4):e1007697. doi: 10.1371/journal.pcbi.1007697 (PMC7173936; doi:10.1371/journal.pcbi.1007697)
Supplement: S3 Fig — Accuracy C vs attention limit k calculated for the whole system (a), uninformed individuals (b) and informed individuals (c). Solid lines are for the interacting system and dashed dotted lines are for the non-interacting system with γs = 0. (PDF) [file pcbi.1007697.s008.pdf]

SUPPLEMENTARY FIGURE 3

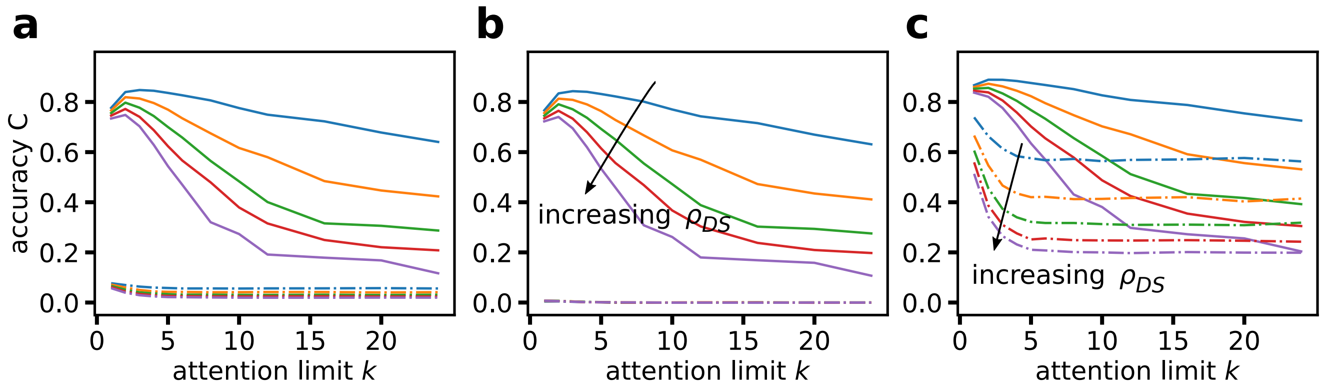

FIG. S3. Emergent collective behavior. Accuracy C vs attention limit k calculated for the whole system (**a**), uninformed individuals (**b**) and informed individuals (**c**). Solid lines are for the interacting system and dashed dotted lines are for the non-interacting system with $\gamma_s = 0$.
