# Supplementary material for: Flocking in complex environments—Attention trade-offs in collective information processing
Source: PLoS Comput Biol. 2020 Apr 6;16(4):e1007697. doi: 10.1371/journal.pcbi.1007697 (PMC7173936; doi:10.1371/journal.pcbi.1007697)
Supplement: S4 Fig — Accuracy C (triangles) and DS avoidance A (circles) versus attention limit k (a). The horizontal line, A = 1, corresponds to DS avoidance of non-interacting agents. For socially interacting agents with low k values (k = 1, 2), we observe high accuracy C together with almost complete ignorance towards environmental cues (see S3 Video). By increasing k, more agents start to sense the environment and react to DSs. At high k, the collective behavior is fully determined by the local environmental features: We observe collective rotation along the circular path and complete ignorance of the global migration direction accessible to informed individuals (see S4 Video). This trade-off is shown quantitatively by the global fitness function in panel b versus attention capacity k and relative DS avoidance benefit β. There are two maxima in global fitness, one for low k, β ≪ 1, showing migration accuracy to be beneficial for the group, the other at high k, and β > 1, which indicates higher benefits associated with DS avoidance in comparison to collective accuracy. (PDF) [file pcbi.1007697.s009.pdf]

SUPPLEMENTARY FIGURE 4

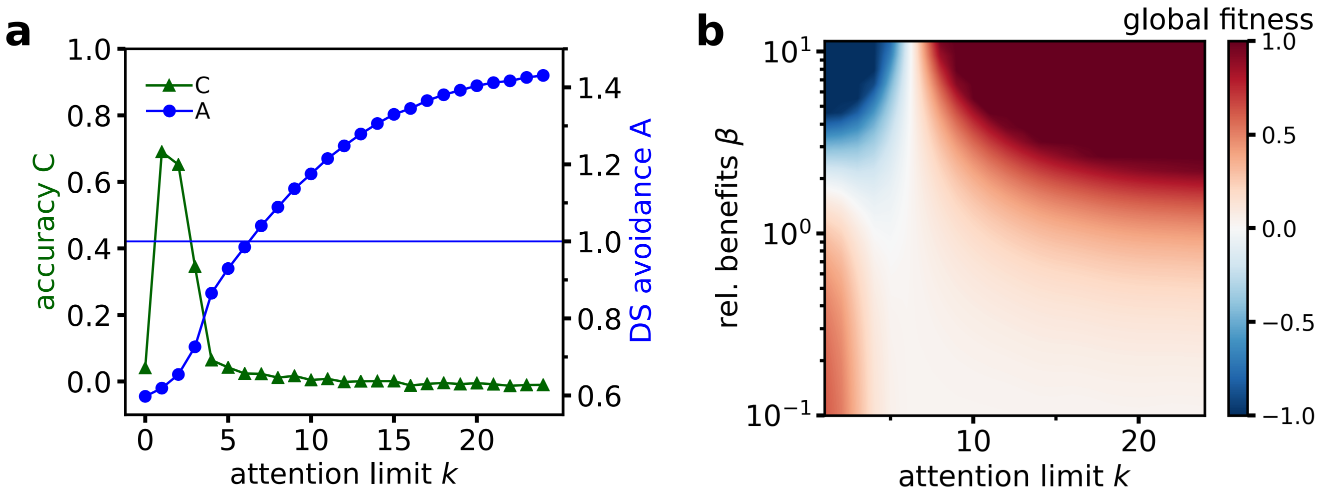

FIG. S4. Collective accuracy and DS avoidance in a structured environment with circular DS free path. **a**: Accuracy C (triangles) and normalized DS avoidance A (circles) versus attention limit k. The horizontal line, $A = 1$, corresponds to DS avoidance of non-interacting agents. For socially interacting agents with low k values ($k = 1, 2$), we observe high accuracy C together with almost complete ignorance towards environmental cues. By increasing k, more agents start to sense the environment and react to DSs. At high k, the collective behavior is fully determined by the local environmental features: We observe collective rotation along the circular path and complete ignorance of the global migration direction accessible to informed individuals (see Supp. Movie S4). This trade-off is shown quantitatively by the global fitness function in panel **b** versus attention capacity k and relative DS avoidance benefit β. There are two maxima in global fitness, one for low k, $\beta \ll 1$, showing migration accuracy to be beneficial for the group, the other at high k, and $\beta > 1$, which indicates higher benefits associated with DS avoidance in comparison to collective accuracy.
