# Supplementary material for: Flocking in complex environments—Attention trade-offs in collective information processing
Source: PLoS Comput Biol. 2020 Apr 6;16(4):e1007697. doi: 10.1371/journal.pcbi.1007697 (PMC7173936; doi:10.1371/journal.pcbi.1007697)
Supplement: S5 Fig — Each agent connected to another individual signaling direct interaction with a DS (direct responder), pays only attention to the signaler(s) and ignores other social cues. Accuracy C (a) and DS avoidance A (b) versus attention limit k for different DS densities at Rinf = 0.1. (PDF) [file pcbi.1007697.s010.pdf]

SUPPLEMENTARY FIGURE 5

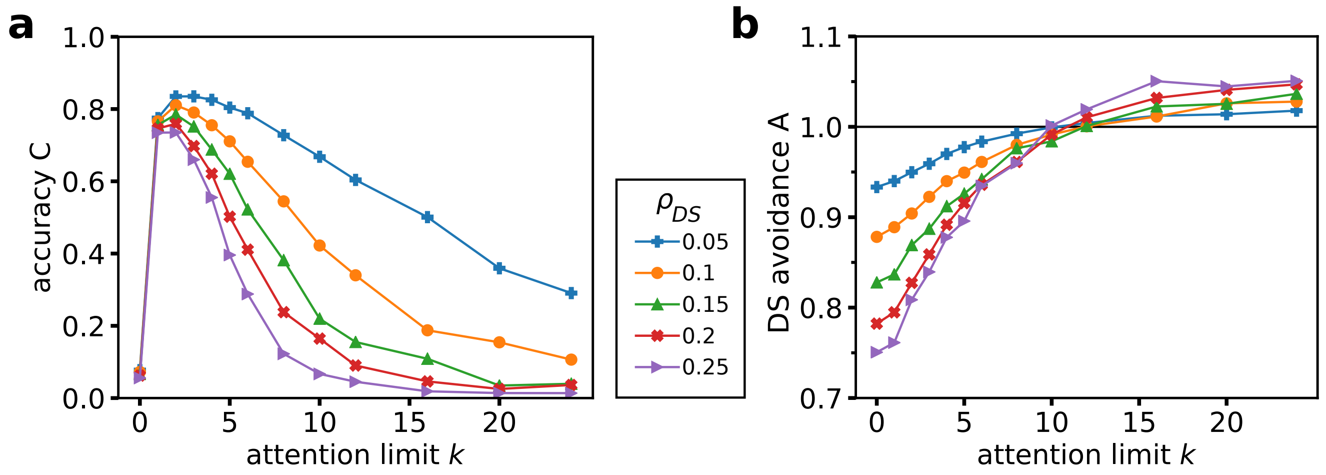

FIG. S5. Attention trade-off in a group of agents with active signalers. Each agent connected to another individual signalling direct interaction with a DS (direct responder), pays only attention to the signaller(s) and ignores other social cues. Accuracy C (**a**) and DS avoidance A (**b**) versus attention limit k for different DS densities at $R_{inf} = 0.1$.
