# Supplementary material for: Flocking in complex environments—Attention trade-offs in collective information processing
Source: PLoS Comput Biol. 2020 Apr 6;16(4):e1007697. doi: 10.1371/journal.pcbi.1007697 (PMC7173936; doi:10.1371/journal.pcbi.1007697)
Supplement: S6 Fig — Accuracy C and DS avoidance A vs attention limit k in a model where the agents first detect DSs with some probability and otherwise interact with their kNO. a: Pdirect = 0.2, b: Pdirect = 0.5. (PDF) [file pcbi.1007697.s011.pdf]

SUPPLEMENTARY FIGURE 6

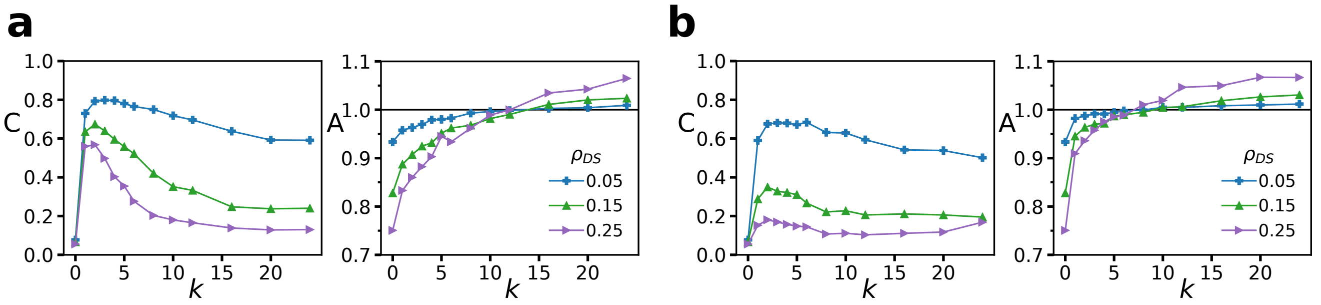

FIG. S6. Model extension with the priority of DS avoidance. Accuracy C and DS avoidance A vs attention limit k in a model where the agents first detect DSs with some probability and otherwise interact with their kNO. **a**: $P_{direct} = 0.2$, **b**: $P_{direct} = 0.5$.
