# Supplementary material for: Flocking in complex environments—Attention trade-offs in collective information processing
Source: PLoS Comput Biol. 2020 Apr 6;16(4):e1007697. doi: 10.1371/journal.pcbi.1007697 (PMC7173936; doi:10.1371/journal.pcbi.1007697)
Supplement: S7 Fig — a: Coordination C˜ (directional order) versus attention limit k for different DS densities. b: DS avoidance versus attention limit k. A = 1 corresponds to non-interacting agents. The qualitative behavior with a coordination-responsiveness trade-off is similar to the model with informed individuals, but here instead of a specific direction, the emergent consensus direction is random (spontaneous symmetry breaking). (PDF) [file pcbi.1007697.s012.pdf]

SUPPLEMENTARY FIGURE 7

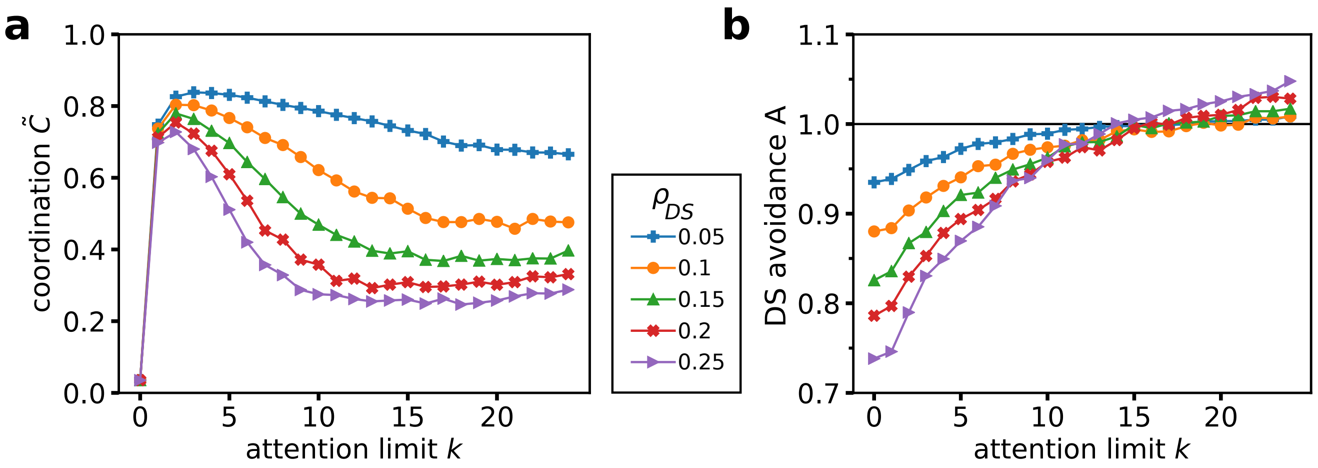

FIG. S7. Emergence of global order in the system with no informed individuals, $R_{inf} = 0$. **a**: Coordination $\tilde{C}$ (directional order) versus attention limit k for different DS densities. **b**: DS avoidance versus attention limit k. $A = 1$ corresponds to non-interacting agents. The qualitative behavior with a coordination-responsiveness trade-off is similar to the model with informed individuals, but here instead of a specific direction, the emergent consensus direction is random (spontaneous symmetry breaking).
