# Supplementary material for: Flocking in complex environments—Attention trade-offs in collective information processing
Source: PLoS Comput Biol. 2020 Apr 6;16(4):e1007697. doi: 10.1371/journal.pcbi.1007697 (PMC7173936; doi:10.1371/journal.pcbi.1007697)
Supplement: S8 Fig — For each focal agent k nearest neighbors are selected from first shell of Voronoi neighbors. If the number of neighbors in first layer is smaller than k, then depending on k, the second Voronoi shell is considered. It is defined by the Voronoi neighborhood of the (direct) Voronoi neighbors of the focal agent. Accuracy C (a) and DS avoidance A (b) versus attention limit k at Rinf = 0.1. (PDF) [file pcbi.1007697.s013.pdf]

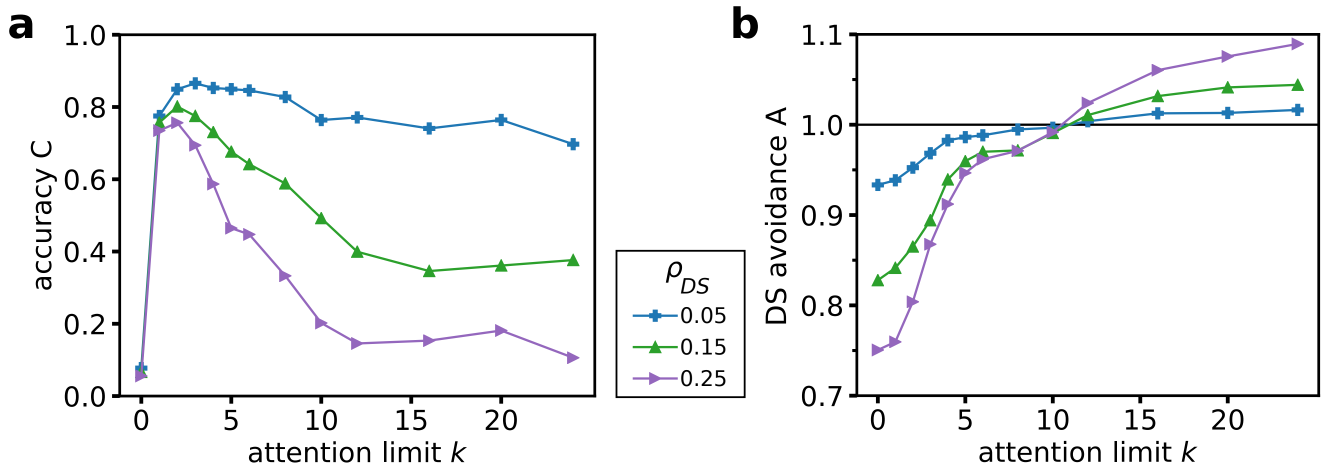

FIG. S8. Collective motion of agents with Voronoi-based kNN interaction network. Here, k nearest agents are selected from first shell of Voronoi neighbors. If the number of neighbors in first layer is smaller than k, then depending on k, the second Voronoi shell is considered. It is defined by the Voronoi neighborhood of the (direct) Voronoi neighbors of the focal agent. Accuracy C (**a**) and DS avoidance A (**b**) versus attention limit k at $R_{inf} = 0.1$.
