# Supplementary material for: Flocking in complex environments—Attention trade-offs in collective information processing
Source: PLoS Comput Biol. 2020 Apr 6;16(4):e1007697. doi: 10.1371/journal.pcbi.1007697 (PMC7173936; doi:10.1371/journal.pcbi.1007697)
Supplement: S9 Fig — The panels show results for changing individual model parameters by a factor of 2 (left columns) and 0.5 (right columns). Different rows represent variation of different parameters, from top to bottom: v0, γs, γl, γp. The non-varied parameters are always set to the default parameters: v0 = 0.5, γs = 1, γl = 1, γp = 0.1. (PDF) [file pcbi.1007697.s014.pdf]

SUPPLEMENTARY FIGURE 9

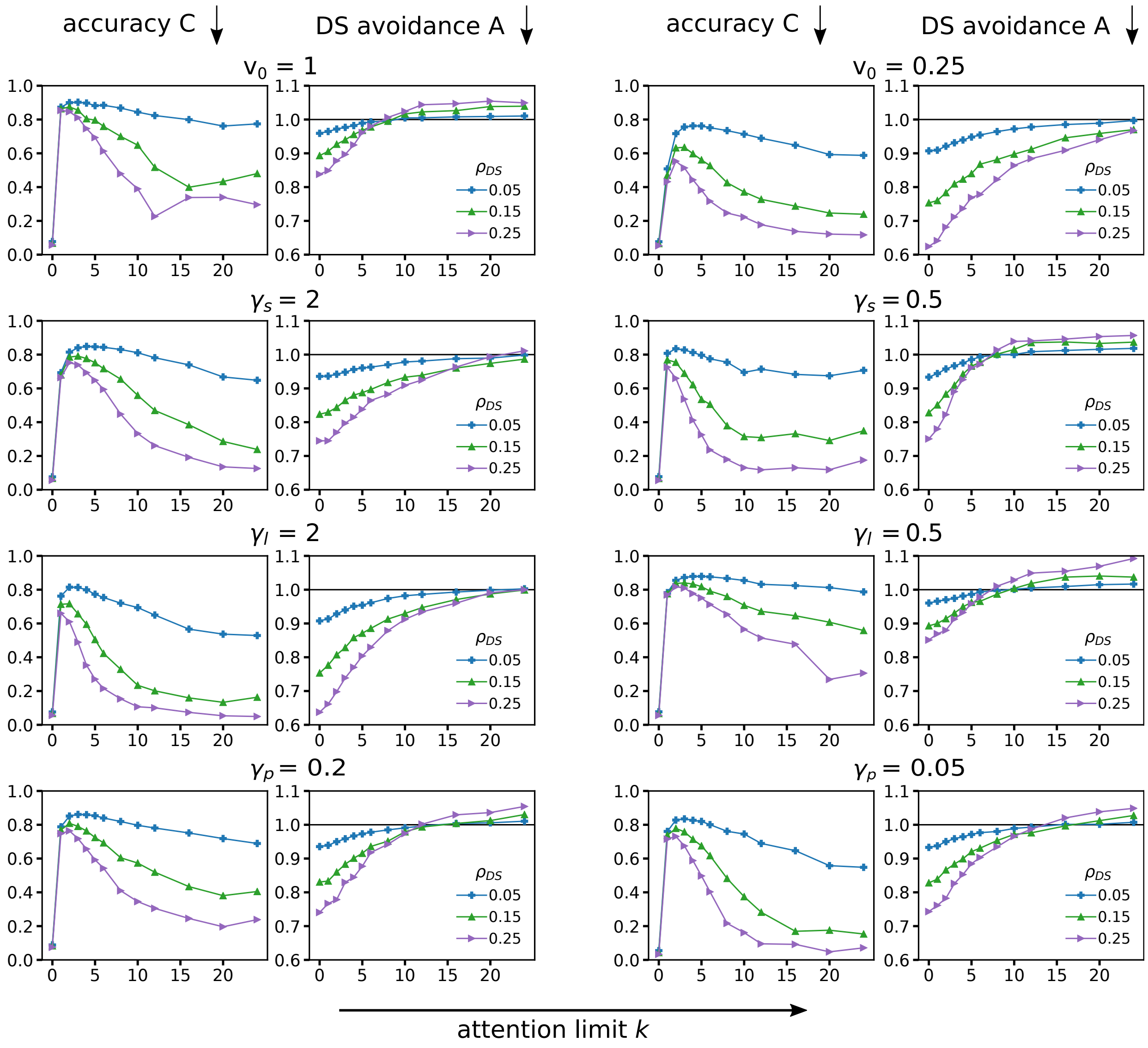

FIG. S9. Robustness of the general results for C and A vs k with respect to variation of model parameters. The panels show results for changing individual model parameters by a factor of 2 (left columns) and 0.5 (right columns). Different rows represent variation of different parameters, from top to bottom: v_0, γ_s, γ_l, γ_p. The non-varied parameters are always set to the default parameters: $v_0 = 0.5$, $\gamma_s = 1$, $\gamma_l = 1$, $\gamma_p = 0.1$.
