# Supplementary material for: Flocking in complex environments—Attention trade-offs in collective information processing
Source: PLoS Comput Biol. 2020 Apr 6;16(4):e1007697. doi: 10.1371/journal.pcbi.1007697 (PMC7173936; doi:10.1371/journal.pcbi.1007697)
Supplement: Attachment — Submitted filename: ResponseReviewers_Rahmani_etal_PLoSCompBiol.pdf [file pcbi.1007697.s015.pdf]

Dear Editor, dear reviewers,

First of all, we apologize for submitting the revised manuscript during the holiday season. However, the deadline for revision was set to Dec 27th and we tried to address the comments by the reviewers as thoroughly as possible within the limited time available.

We thank the referees for the thorough revision and the thoughtful and critical comments on our manuscript. We have made substantial revisions to our manuscript, including additional simulations of an extended model to address the points raised by the reviewers. We are convinced that our revision significantly improved our manuscript and provided even stronger support for our main claims.

Further below we provide replies to all points raised by both referees. Where applicable, we also provide information on the corresponding revisions to the main text and supplementary information. We submit, for both the manuscript and the supp. information, a clean version as well as a file with changes marked in color.

We hope that with our revisions and responses we can satisfy both referees and that our manuscript will be acceptable for publications with PLoS Computational Biology.

With best regards

Pawel Romanczuk, Parisa Rahmani, Fernando Peruani

Detailed Response to Referee comments

Reviewer #1:

The authors studied a Vicsek like model of flocking in environments with obstacles. The novelty of the model is placing a cap on the number of interacting neighbors (k). The model is studied systematically under different density of avoidance zones and values of k, and reveals a fairly unintuitive result: for a wide range of obstacle densities, systems with low k's achieve higher accuracy than systems with larger k values.

Overall I find this to be an excellent paper: the topic is of wide interest, the text is well written, and the methods are sound. However, I do have a few comments that I hope the authors could address:

(1) This is a minor one. The terms "heterogeneous environments" and "homogeneous environments" are frequently used in the text, but they are not well defined. It would be worthwhile mentioning in the introduction that you will be referring to simulation with obstacles (or distraction sites) as "heterogeneous environments".

We thank the referee to point out the lack of explicit definition of “heterogeneous” versus “homogeneous” environments. We note that we refer to “complex environments” in the abstract and introduction, which in the context of the paper is equivalent to “heterogeneous” environments. We added a corresponding clarification/definition of the terms in the text at line 56.

(2) The authors define the accuracy of the collective migration as the average deviation from the preferred direction of motion u_p. But they do not take into account the avoidance of obstacles in their accuracy measure. Wouldn't avoiding those "danger zones" be beneficial and even crucial for the success of the flock? I suggest to include a discussion of the success of avoiding the obstacles.

We distinguish in our paper explicitly the “migration accuracy”, or just short “accuracy”, from obstacle avoidance efficiency, which is measured through the “obstacle avoidance” variable (see Fig. 4b). Through proper normalization of obstacle avoidance we account for the trivial effects of an increasing obstacle density, and are able to compare the performance of the collective relative to solitary agents (see Methods “Avoidance Parameter”). We combine both measures in the fitness function as shown in Fig. 4c and discussed in Methods (“Fitness Function” section). However as both measures: accuracy and obstacle avoidance are

fundamentally different, i.e. they are measured on different, non-comparable scales, there remains the question of how to weight them relative to each other when calculating a combined measure (fitness). This is why we need to consider an additional parameter, the relative benefit of obstacle avoidance with respect to migration accuracy to account for possible different ecological scenarios.

However, based on the referee comment we acknowledged that in the Discussion section there was a bias towards discussing only the migration accuracy. We revised the discussion to more prominently discuss the efficiency of obstacle avoidance in the context of the attention-responsiveness trade-off (lines 348-355) .

(3) By setting the maximal number of neighbors an individual can pay attention to, the simulations are prone to large fluctuations in the lists of interacting neighbors. Depending on the configuration, an individual could switch attention from one neighbor to another, even if those neighbors are separated by a large spatial distance. This should be mentioned somewhere in the text, and preferably quantified somewhere in the SI.

Indeed this is what can happen in the model. However, this is not a feature of the kNN-model alone. This happens in the same way in metric models with a fixed interaction radius. An agent can lose neighbors e.g. by moving forward and leaving them behind while gaining new neighbours up front. Or by slight turns losing neighbors on one side and gaining new ones on the other side. Also there the two agents may be separated by large distances. However, in metric models this maximal separation is bounded to 2R with R being the interaction range, for kNN this is in principle unbounded, yet we observe in heterogeneous environments formation of dense clusters. Thus, in our case the maximal separation is effectively limited to the cluster size. We have added a corresponding discussion in the SI Appendix 1.

In the SI we had already Fig. S2c showing the average edge life time, and its dependence on obstacle density and attention capacity. Following the referee's suggestion, we added a brief section discussing the dynamics of the interaction network, as well as an additional subfigure (Fig. S2b) showing the probability to observe a single connected component (see comment below).

(4) When using high k values (say 24) and high obstacle densities (say 0.25), it's not clear that it is possible to obtain a line of site with all the interacting neighbors. The authors could check this in their simulations, and limit reporting results only to more physically realistic regimes.

We acknowledge the concern by the referee. However, we should note that our model - as any theoretical model - is an idealization of the behavior of natural systems. The main purpose of the study was to systematically investigate the question on how the shared attention to social and environmental cues modifies the emergent collective behavior at the macroscopic scale. For clarity and simplicity we focused on a agent-based model, which 1) is as simple as possible

and 2) represents a natural “first order “ expansion of existing models towards non-empty environments.

Specifically in order to keep the model as simple as possible we refrained from considering finite size agents and distraction sites. This would require a whole new set of modeling decisions e.g. on the actual physical interaction of agents and finite size “obstacles” (excluded volume) in addition to possible repulsion zone around them. Thus we explicitly assume point-like agent and point-like environmental cues (“distraction sites”), which are surrounded by danger zones which itself represent the areas where an agent responding to the cue attempts to move away from it as fast as possible. Therefore, line of sight obstruction are mathematically speaking not possible in this model. This idealizing assumptions is reasonable in cases where the actual physical dimensions of the agents and environmental cues are small compared to the typical interaction ranges. Furthermore, we consider here for simplicity a two-dimensional system which in itself is an idealization. Real collective behavior in nature happens in three dimensions whether its aquatic systems (fish schooling) or terrestrial systems (ungulate herds). In 3d dimensions, even for finite size objects blocking the line of sight becomes much harder if we consider not to dense environments, e.g. antilopes can look above suspicious grass or smaller bush structures.

Furthermore, for the KNN-interaction network, the rank of nearest objects is intrinsically related to their distance, and in particular for high k we observe the formation of dense groups. Thus, the situation where one of the neighbors is within the KNN-set and at the same time “behind” a distraction site is extremely rare.

Last but not least, and probably most importantly, we considered also a Voronoi variant of our model, which corresponds to a spatially balanced KNN-model, where obstructions become even more improbable. The qualitative results remain unchanged (see SI Fig. S8).

Therefore, we are convinced that taking such line-of-sight obstructions into account does not change the findings of our paper. However, we hope that our first model will motivate further studies which consider additional physical constraints (e.g. excluded volume) to investigate how this modifies our general findings.

Based on the referee comment, we have added a new Appendix (#6) section with the above discussion of the idealizing assumptions of our model.

Reviewer #2:

Rahmani et al. introduce a model of collective motion with limited agent perception in spaces with danger areas. This model builds on well studied examples from the literature, in particular the flocking models of Vicsek and colleagues, using a topological interaction regime limited to k nearest-neighbours or danger sites. They also incorporate ‘informed individuals’ who have a preferred direction of motion. They study the collective motion of agents in this model as a function of the attention capacity (k) as well as the density of danger sites and the proportion of informed individuals. The key result is that the collective motion displays somewhat counter

intuitive properties as the attention capacity is increased: group cohesion and accuracy breaks down as agents are driven primarily to avoid danger sites.

I found the main result of this paper interesting and potentially important, but I have concerns about its significance from both a biological and an analytical perspective.

Analytically, I think the authors could do more to demonstrate the observed effects are genuinely an emergent collective phenomenon. In particular, if we were to consider a model with a zero-strength social interaction, we would expect to see that 'collective accuracy' would be decreased as k increases as more and more informed individuals are disrupted, while uninformed individuals would experience no change from their random orientations. To what extent do these results go beyond this expectation? There is a partial answer to this in Figure 4 A, but I would have found it useful to see a breakdown in the effect on informed and uninformed individuals separately.

We included the results for the model with zero social strength (gamma_s=0) into the SI together with the original model (gamma_s > 0) as a comparison (see Appendix 2 and Fig. S3). In Fig. S3a (accuracy of the whole flock vs k) it is clearly observed that the group accuracy as a whole is negligible in the absence of social interactions. However as the referee points out the effect would be different for informed and uninformed individuals if we consider their accuracy separately. Uninformed individuals move randomly in the absence of interactions while with social interactions they reach accuracy which decreases with increasing k (Fig. S3b accuracy of uninformed individuals vs k). Whereas informed individuals have high accuracy at low k which quickly saturates to a constant value, therefore their behavior is different from the decreasing trend observed in the social version of the model (Fig. S3c accuracy of informed individuals vs k). We should also notice to the rise in the accuracy of informed individuals in the social model compared to non-social model, which is due to social interactions with uninformed individuals.

We can approach the problem of genuinity of collective behavior from another perspective by noting that the qualitative behavior of the accuracy measure is even observed for zero informed individuals, when "accuracy" is replaced by "migration consensus" (see main text lines 316-324 and SI Fig. S7). Therefore, regardless of the behavior of informed individuals, collective tend to be more accurate at low k values relative to higher k values.

Last but not least, the "avoidance" parameter itself is a relative measure, and it is computed relative to a system of non-interacting agents. A vs k curve (see Fig. 4b) clearly shows collectives outperform solitary non-interacting agents in only high k values and fall below it in low k values indicating that this behavior also emerges from social interactions.

I was also unable to locate a justification for the assigned values of many model parameters, or a detailed discussion of how sensitive the model may be to these. For instance, L419 notes that the results are qualitatively unchanged under variation of v_0, eta and L, but does not mention variations in the gamma parameters or in the density of agents (N/L^2)

We extended that part (lines 463-466) to explain the behavior of the model with changing different model parameters, and included the results for some of these variations in SI Fig. S9 .

Biologically my concern focuses on how realistically the model represents real systems of interest. Obviously the model is a theoretical abstraction, and absolute realism is neither expected nor desirable. However, one feature of the model struck me as possibly unrealistic: agents treat danger sites on an equal footing with other agents when allocating attention (i.e. a danger site must be within the k nearest objects to be attended to, even if one is within the danger radius), yet once the agent does attend to a danger site this overrides any social response. This seems inconsistent: if danger sites are overwhelmingly important to attend to, surely the agents ought to be more active in attending to their possible existence. The only explanation I could conceive of to explain the equal treatment is if interactions are visual, and attention is moderated by visual occlusion (and agents make no significant effort to overcome occlusion). I think the authors should discuss this assumption explicitly in the paper and potentially investigate the robustness of their results to alternative ways of allocating the limited attention. As it stands and based on this model alone, I can't agree with the authors' assertion that this 'demonstrates a fundamental trade-off' (L313-314).

Here we consider mainly visual information in the sense that objects should be perceived/seen in order to become influential. And perceiving in our model is defined by being in k nearest objects, i.e. nearest objects are perceived first. In other words, the weight for observing DSs is equal to the weight of seeing agents, and the one that is closer becomes impactful. Therefore, it might happen that the focal agent is on the DS repulsion zone and is not aware of it because has not perceived it.

In the absence of specific empirical systems and results where relative attention allocation has been measured, the assumption of equal attentional weights is perfectly justified. However, we acknowledge the concern by the referee that for biological agents there might be a bias of attention towards environmental cues if they may signal danger, and that this may modify our findings. In order to demonstrate that our general findings hold even if there is a higher weight assigned to the environmental cues, we devised a model extension (see Appendix 4) where the attention to nearby objects is allocated differently by putting a higher priority on DS detection. In the extended model, we introduce an additional parameter P_{direct}, which represents the probability that the agent will detect the DS once it is within the avoidance zone, independently of its social neighborhood, i.e. independently whether it is within its kNO. For $P_{direct}=0$ the extended model reduces to the original model assuming equal detection of social and environmental cues, as discussed in the main text. For $P_{direct}=1$ the agents deterministically detect the DS once within the avoidance zone. Our qualitative result on the attention-responsiveness trade-off remains valid as long as $P_{direct}<1$ (See SI Fig. S6). Thus as long as there is some sensory "interference" between the response to environment and response to social cues, which is reasonable to assume for agents with limited sensory and cognitive capability, there will be a corresponding trade-off. In summary we show that it is justified to speak about a "fundamental trade-off".

In the discussion the authors point to several empirical studies in support of their central finding. I found the connection with ref. 43 (Guinea baboons) to be unconvincing as a comparison here.

This example was proposed by Prof. Julia Fischer, the senior author in [43], during a talk and a subsequent private communication. Guinea baboons living in complex social environments (multiple groups, in near spatial vicinity) appear to filter distracting social cues by limiting their attention only to sounds produced by members of the same social group, i.e. neighbors in the self-organized social-network. We included this as the general principle maps directly one-to-one mapped to our observation on the flocking movement in real space. We revised the paragraph to emphasize the speculative nature of this abstraction.

The authors point to ref 21 (Jiang et al) as an empirical example of fish attending to a limited set of neighbours, however the abstract of this paper states ‘we find no correlation between the distance rank of a neighbor and its likelihood to be influential’, which does not align with the model used here.

Jiang et al was cited because of the demonstration that fish appear to interact with only 1 or 2 neighbors at a time. Indeed, the study does not a significant correlation of the distance rank with influence, albeit there is a weak effect. However this is a very special experimental scenario, quasi 1d, only considering u-turns (reversal of motion) in small highly cohesive groups of up to N=5. Also the distance rank is not equivalent to actual distance. However in general distance dependence of social interactions has been repeatedly confirmed in fish see e.g. [Katz et al, PNAS 108, 2011, Herbert-Read et al, PNAS 108, 2011, Rosenthal et al PNAS 112, 2015] and is an accepted assumption of all flocking models, as it otherwise leads to unphysical and unbiological results of unbounded interaction ranges. Thus it can not be excluded that the lack of distance dependence in Jiang et al, is a consequence of the specific experimental set-up and context.

I am also skeptical about the speculation regarding robot swarms. There seems to me no reason why perception itself need be limited in agents to produce cohesion and other desirable effects: agents could be programmed to respond to fewer stimuli than they can perceive, or to intelligently to different sources of information. I think the authors are on stronger ground when they state that their results generate predictions and testable hypotheses, and I would suggest that more focus is given to laying these out in some detail and discussing how they could be tested.

We agree with the referee that with swarm robots with camera one does not need to limit the sensory capabilities. What we suggested and meant is actually what the referee writes:

(...) agents could be programmed to respond to fewer stimuli than they can perceive, or to intelligently to different sources of information.

This is exactly the point we are making: Instead of programming complex algorithms for classifications of visual cues (“Is this an environmental cue or another agent?”), e.g. computationally intensive, data-hungry machine learning algorithms, one can instead tailor specific attentional bottlenecks as motivated by the limited attention to visual stimuli of social animals including humans.

Furthermore, we note that Kilobots as the only truly scalable swarm robotics platform in the world, have very limited computational abilities and sensors. Any truly scalable swarm robotics platform in the future, will need to keep the single agents as simple as possible. Thus it will be far behind the current state-of-art in terms of sensors due to limited computation and battery life times of single agents. Our paper shows that such sensory limitations might surprisingly be even beneficial for movement coordination in complex environments if used wisely.

Minor points:

1. Would it better to define the density of danger sites as the actual proportion of space taken up by danger sites rather than the number of danger sites divided by L^2? For one thing, this would make results more comparable across different values of L.
The number of danger sites could be easily tuned while the proportion of space could not be tuned easily, especially because of the circular nature of danger sites and the fact that we allow them to overlap. But each data point is the average of 20 realizations, meaning that the effect due to having different surface fractions is washed out to some extent, and we are left with the average behavior.

2. Panels C and D of Figure 2 do not seem to be discussed anywhere in the main text.
Actually they do appear in the maint text (lines 188 and 201 in the revised version), but the order of references to some subplots in figures is not according to the order that they appear in the text, since we wanted to have some related plots in one figure.

3. The authors repeatedly refer to the effect of attention on collective ‘consensus’ (e.g L176), but the results pertain to collective ‘accuracy’. These are not the same; if all agents move coherently perpendicular to the preferred direction they would have consensus but not accuracy.
In our model with informed individual consensus forms always in the preferred direction. In the model without informed individuals, instead of accuracy (alignment with the preferred direction of motion) one has to consider (directional) consensus (alignment with the random average direction of motion). The results for both variables versus k are basically the same as discussed towards the end of the results section and SI Fig. S7. Thus directional consensus and accuracy are essentially equivalent as discussed. However, we agree that this might be confusing so we made minor changes in lines 179, 207 (replacing consensus by accuracy) and added an explaining sentence pointing out the equivalence in line 227ff.

4. The use of delta to parameterise the relative benefits of danger site avoidance in equation 5 was confusing to me, as it looked like a dirac delta function. I would suggest using something less ambiguous.
We have changed the symbol for the corresponding parameter (ratio of benefits) to \beta in order to avoid this possible misunderstanding.

5. L137 - what is the 'critical value' to create a connected graph? Is it consistent across simulations, and can it be compared to the typical value needed to connect uniformly scattered points (c. 3-4 in 2D I think)?
We are not entirely sure what the referee means by the value of 3-4 in 2D, it seems that it may refer to the percolation threshold in spatial kNN-graphs [Balister, P., et al. In Handbook of large-scale random networks. Springer, 2008.] where the authors show with high confidence that k= 3 is the actual threshold for percolation, i.e. the probability of observing infinite CC is nonzero, see also [Teng, S.H. and Yao, F.F. Algorithmica 49, 2007.]. However, percolation is not exactly the same as a fully connected graph (although certainly related). Percolation indicates the formation of a giant connected component where for N->∞ the relative mass of the disconnected nodes vanishes. However, we acknowledge the referee's suggestion to be more quantitative about the transition to fully connected networks and we added a corresponding brief discussion in the SI Appendix 1 as well as an additional subfigure in Fig. S2 (panel b).

As we consider finite, time-varying graphs, in order to deduce the critical value, we calculated the probability of observing one CC during simulations, and included it in SI (Fig. S2b) to make our arguments about connectivity more quantitative. In the plots it is observed that nonzero probability happens around k=8 for the lowest DS density, and increases to the value of around k=20 for the largest DS density which is not comparable to the uniformly scattered points due to heterogeneity in the environment and its impact on the social interaction network.

6. L263, should be dynamics ARE not dynamics IS
We thank the referee for identifying this. It has been corrected.

7. In equation 5 the costs/benefits associated with accuracy and avoidance and assumed to be linear. Can this be justified biologically, or is this just a convenience?

In our work, linear fitness function is the simplest choice which can be used to quantify the trade-off observed in our data. Potentially many other functional forms can be assumed which will also show the two distinct maxima. Here, in the absence of any good reason to assume a different functional form we went with a linear superposition.
